# The Occurrence of *Cryptosporidium* spp. in Wild-Living Carnivores in Poland—A Question Concerning Its Host Specificity

**DOI:** 10.3390/pathogens12020198

**Published:** 2023-01-28

**Authors:** Agnieszka Perec-Matysiak, Joanna Hildebrand, Marcin Popiołek, Katarzyna Buńkowska-Gawlik

**Affiliations:** Department of Parasitology, Faculty of Biological Sciences, University of Wrocław, Przybyszewskiego 63, 51-148 Wrocław, Poland

**Keywords:** *Cryptosporidium* spp., wild-living carnivores, invasive species, zoonosis

## Abstract

*Cryptosporidium* is an apicomplexan protozoan parasite that primarily infects the gastrointestinal epithelium in humans and domestic and wild animals. The majority of studies have been focused on human, livestock, and pet infections. Hence, *Cryptosporidium* spp. in wildlife, including wild carnivores, remained neglected. There are several studies reporting the occurrence of *Cryptosporidium* spp. in wild foxes, but these are only a few molecular surveys; no data is available concerning the occurrence of this parasite in raccoon dogs and martens in Europe, and to the best of our knowledge to date, only one study has reported *Cryptosporidium* from badgers in Spain. Therefore, we used molecular analyses to identify and genotype *Cryptosporidium* spp. in wild-living mesocarnivores in Poland. A total of 322 individual fecal samples from six carnivore species, i.e., raccoon, raccoon dog, red fox, European badger, pine, and beech martens were collected and then analyzed for the presence of *Cryptosporidium* spp. using the nested PCR method. The appearance of PCR products in the reaction with *Cryptosporidium*-specific primers against the 18S rRNA and actin genes demonstrated that *Cryptosporidium* spp. occurred in 23.0% of all examined species of animals. Performed sequence analyses showed the presence of the *Cryptosporidium* skunk genotype, *Cryptosporidium* vole genotype II, *Cryptosporidium canis* dog and fox genotypes, as well as *Cryptosporidium erinacei*, *Cryptosporidium ditrichi*, *Cryptosporidium suis,* and *Cryptosporidium alticolis,* in these hosts. Molecular data presented here indicate that examined mesocarnivores may be a significant reservoir of specific and non-specific *Cryptosporidium* species, including those with zoonotic potential. Most studies of carnivores have described the presence of non-specific *Cryptosporidium* spp. in carnivore hosts, and this is probably the result of the transfer of these parasites from prey species through the digestive tract or the transfer of the parasite from a contaminated environment.

## 1. Introduction

*Cryptosporidium* is an apicomplexan protozoan parasite that primarily infects the gastrointestinal epithelium in humans and domestic and wild animals [1]. Due to recent progress in molecular diagnostic techniques, of the 44 *Cryptosporidium* species and over 120 genotypes, 19 species, and 4 genotypes have been reported in humans. However, *Cryptosporidium hominis* and *Cryptosporidium parvum* account for 95% of human infections, followed by *Cryptosporidium meleagridis*, *Cryptosporidium felis,* and *Cryptosporidium canis* [2,3]. A recent study from Poland has documented a direct animal-to-human transmission of an unusual subtype of *Cryptosporidium* sp. horse genotype in an immunocompromised patient [4]. The majority of studies are focused on human, livestock, and pet infections. Thus, *Cryptosporidium* spp. in wildlife, including wild carnivores, remain neglected [5].

A great diversity of *Cryptosporidium* species/genotypes has been found in mesocarnivores belonging to Procyonidae, Canidae, and Mustelidae living in the wild in Spain, Ireland, Poland, Germany, the Czech Republic, Slovakia, Iran, Japan, and the U.S.A. [6,7,8,9,10,11,12,13,14,15]. Recent molecular research has identified *Cryptosporidium* spp. in farmed fur animals (foxes and raccoon dogs) in China [16,17,18]. During molecular studies, the zoonotic *Cryptosporidium* spp. has been recorded in these hosts, i.e., *C. hominis*, *C. parvum*, *C. meleagridis*, *C. andersoni*, *C. canis*, *C. felis*, *C. ubiquitum*, *C. erinacei*, *C. tyzzeri*, *C. suis*, *C. scrofarum*, *C. ditrichi,* and the *Cryptosporidium* skunk genotype.

The occasional occurrence of some of the *Cryptosporidium* species in humans suggests that wild carnivores contribute to environmental contamination with potentially zoonotic *Cryptosporidium* spp. [17,19,20]. Thus, the occurrence of a variety of zoonotic pathogens in wild animals raises a number of issues with major implications for domestic animals and human welfare. Carnivore species, both introduced and invasive, such as the raccoon and raccoon dog, as well as domestic species such as the red fox, badger, and marten, are widely found throughout Europe, including Poland.

The presence of raccoons near human settlements may pose a threat to human health because, as research conducted in the United States has shown, this predator is the host of a broad range of parasites and pathogens that are dangerous to humans and domestic and wild animals [21]. Studies of raccoons that were introduced to Europe have so far revealed the presence of zoonotic genotypes of *Cryptosporidium* [10,13]. Another introduced species, the raccoon dog, may serve as a reservoir for zoonotic agents that affect native co-occurring species such as the red fox [22,23], badger, and martens. The red fox is the most common wild canid in Europe, with a high population density and wide geographical distribution, including highly urbanized areas [20,24,25].

There are several molecular studies reporting the occurrence of *Cryptosporidium* spp. in wild-living carnivores in Europe. There are a limited number of molecular surveys concerning foxes and raccoons. No data are available on the occurrence of this parasite in raccoon dogs and martens, and to the best of our knowledge to date, only one study has reported *Cryptosporidium* in badgers from Spain [11], despite their wide distribution and population density. Therefore, we used molecular analyses to identify and genotype *Cryptosporidium* spp. in wild-living raccoons, raccoon dogs, red foxes, badgers, and martens in Poland. In addition, we assessed these mesocarnivores as a significant reservoir of specific and non-specific *Cryptosporidium* species, including those with zoonotic potential.

## 2. Materials and Methods

### 2.1. Study Areas and Specimen Collection

**Figure 1:**
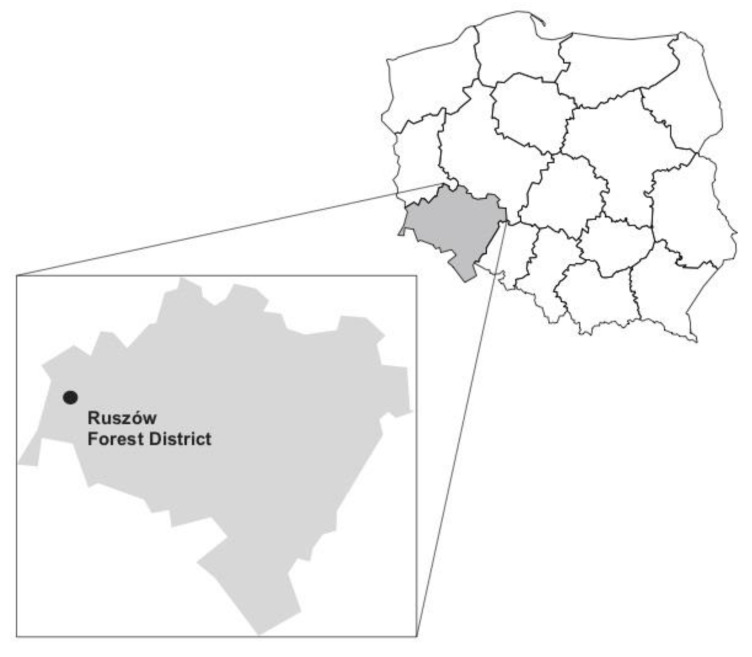
The map of Poland showing geographical origin (black dot) of wild carnivores obtained for this study.

In the materials for this study, a total of 322 fecal samples from 6 carnivore species (raccoon (*Procyon lotor*; n = 65), raccoon dog (*Nyctereutes procyonides;* n = 87)*,* red fox (*Vulpes vulpes*; n = 50), European badger (*Meles meles*; n = 45), pine martens (*Martes martes*; n = 24), and beech martens (*Martes foina;* n = 51)) were collected during the period of 2017–2019. All fecal samples were obtained during necropsy from animals shot during a predator control operation or road-killed animals from Ruszów Forestry (51°24′00.1″ N 15°10′12.2″ E), Bory Dolnośląskie, the Lower Silesian District in Poland, where, uniquely, the invasive and native carnivore species co-occur in the same habitat (Figure 1). All samples were kept at −20 °C until further analysis.

### 2.2. DNA Extraction

DNA was extracted from the fecal samples using the GeneMATRIX Stool DNA Purification Kit (EURx, Gdańsk, Poland) according to the manufacturer’s protocols. The DNA isolates obtained were stored at −20 °C until molecular analysis was completed.

### 2.3. Molecular Detection and Genotyping of Cryptosporidium *spp*.

The molecular detection and genotyping of *Cryptosporidium* spp. was performed in two steps, i.e., (1) nested PCRs targeting the 18S rRNA gene on fecal samples of examined carnivore species (detection method, high sensitivity); (2) nested PCRs targeting the actin gene on samples with a positive result in the 18S rRNA PCR (genotyping method used for the sequence analyses and phylogeny due to actin genetic variability).

In the first step PCR amplification was performed using sets of nested primers amplifying 18S rRNA gene of *Cryptosporidium* spp. with cycling parameters elaborated by Xiao et al. (1999) [26]. PCR amplification was performed in a T100 Thermal Cycler (BioRad) on a set of primers, i.e., 5′-TTCTAGAGCTAATACATGCG-3′ and 5′-CCCTAATCCTTCGAAACAGGA-3′, and 3 μL template DNA for the first reaction. For the secondary PCR step, a PCR product (819 to 825 bp) was amplified by using primers 5′-GGAAGGGTTGTATTTATTAGATAAAG-3′ and 5′-AAGGAGTAAGGAACAACCTCCA-3′, and 2 μL of the primary PCR product. Then it isolates with a positive result in 18S rRNA-PCR. We performed the nested-PCR for actin gene according to protocol, elaborated by Sulaiman et al. (2002) [27]. PCR amplification was performed using forward (5′-ATG(A/G)G(A/T)GAAGAAG(A/T)A(A/G)(C/T)(A/T)CAAGC-3′) and reverse (5′- AGAA(G/A)CA(C/T)TTTCTGTG(T/G)ACAAT-3′) primers, and 2 μL template DNA for the first reaction. For the secondary PCR, a fragment of ~1,066 bp was amplified using 1 μL of product from primary PCR and a set of primers i.e., 5′-CAAAGC(A/T)TT(G/A)GTTGTTGA(T/C)AA-3′ and 5′-TTTCTGTG(T/G)ACAAT(A/T)(G/C)(A/T)TGG-3′. All PCR amplifications were performed in 25 μL reaction volume, consisting of 12.5 μL of the standard and ready-to-use PCR mixture 2xPCR Mix Plus (A@A Biotechnology, Gdańsk, Poland), 0.25 μL of each primer (10 mM), and DNA and respective volume of ddH_2_O. For all PCR reactions, negative and positive controls were performed using sterile water and reference DNA, respectively.

Secondary PCR products were resolved by electrophoresis in a 1.0% agarose gel and stained with Simply Safe (EURx, Gdańsk, Poland) (Figures S1 and S2). Products of the expected size were purified using a QIAquick PCR Purification Kit (Qiagen, Hilden, Germany) and stored at 4 °C until sequenced.

### 2.4. Sequence and Phylogenetic Analysis

Products were sequenced in both directions using an Applied Biosystems ABI PRISM 3100-Avant Sequencer (SEQme, Dobříš, the Czech Republic). The nucleotide sequences obtained were manually edited with the DNA Baser Sequence Assembly software (Heracle BioSoft SRL, Mioveni, Romania) and aligned with reference sequences of *Cryptosporidium* spp. available in GenBank. Phylogenetic analyses were performed using the MEGAX software [28]; a tree was constructed using the Maximum Likelihood (ML) (GTR + G + I model); and bootstrapping was performed using 1000 replicates. Sequences obtained in this study were deposited in the GenBank database under the accession numbers KX639723, MK241550, MN237647-MN237649, and MK248707-MK248711.

### 2.5. Statistics

The prevalence of *Cryptosporidium* spp. was evaluated based on the positive PCR results of the stool samples. The 95% confidence interval (CI) was calculated for each prevalence.

## 3. Results

The fecal samples obtained from 322 native and invasive mesocarnivore species were analyzed for the presence of *Cryptosporidium* spp. using the nested PCR method (Table 1). The appearance of the PCR product in the reaction with *Cryptosporidium*-specific primers against the 18S rRNA and actin genes demonstrated that *Cryptosporidium* spp. occurred in all examined species of animals, showing the overall *Cryptosporidium* prevalence at a level of 23.0%. Both of the examined invasive carnivores showed *Cryptosporidium* prevalence at similar levels (24.6% for raccoons and 24.1% for raccoon dogs). *Cryptosporidium* infection rates among native carnivores were noted to be the highest for martens (29.2% for pine martens and 29.4% for beech martens) and the lowest for red foxes (12.0%). We sequenced all actin amplification products (74 amplicons), and analysis of the variable actin locus, was employed in the phylogeny. Our data for actin sequences derived from particular isolates showed *Cryptosporidium* species and genotypes identified in these hosts (Table 1). Selected 18S rRNA products (12 amplicons) were sequenced to verify the specific amplification of the *Cryptosporidium* spp.

**Figure 2:**
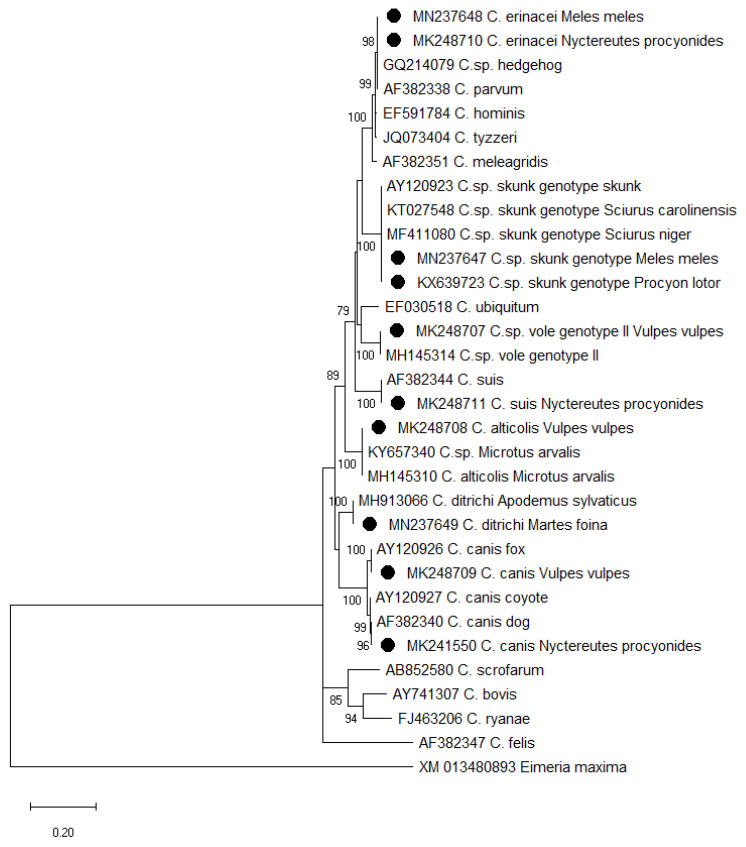
The phylogenetic relationship of *Cryptosporidium* spp. identified in this study and others as inferred by the Maximum Likelihood method of the partial actin gene sequences. Bootstrapping was performed using 1000 replicates; values below 75% are not shown. Sequences from this study are marked with solid circles.

The phylogenetic analysis of the actin gene in *Cryptosporidium* skunk genotype positive raccoons and badgers showed the identity of the isolates obtained from raccoons from Poland (our earlier report [10]) and from the Eastern fox squirrel *Sciurus niger* naturally occurring in Canada (KT027548) as well as showing 99.9% and 99.8% homology with the sequence obtained from the grey squirrel *Sciurus carolinensis* from Italy (MF411080) and the skunk *Mephitis mephitis* from the U.S.A. (AY120923), respectively (Figure 2).

The analysis of the actin gene in *C. canis*-positive raccoon dog isolates and the *C. canis*-positive red fox isolates showed identity with the *C. canis* dog genotype (Acc. No. AF382340) and *C. canis* fox genotype (AY1209260), respectively. The sequences from one isolate derived from a raccoon dog and from one isolate obtained from a badger showed 99.9% (998/999bp) homology with *C. erinacei* (previously hedgehog genotype GQ214079). The other actin sequence obtained from the raccoon dog was identical with *Cryptosporidum suis* (AF382344). For the three remaining isolates from foxes, one sequence derived from the *Cryptosporidium* actin gene showed 100% identity with the GenBank sequence identified as *Cryptosporidium* vole genotype II (MH145314), and two other nucleotide sequences showed 99.8% (945/947bp) homology with the GenBank sequence described as *Cryptosporidium alticolis* (MH145310). These two last genotypes are specific for common voles (*Microtus arvalis*). The isolates of *Cryptosporidium ditrichi* identified in both species of martens were identical to the sequences identified in *Apodemus sylvaticus* (MH913066) from the Czech Republic. Single nucleotide polymorphism analysis of the obtained 793bp actin sequences for *C. canis* and reference sequences (*C. canis* fox, dog, and coyote genotypes) showed 19 SNPs without insertions/deletions. In the case of other *Cryptosporidium* species, we recorded single SNPs (in the case of the *Cryptosporidium* skunk genotype, *C. erinacei*, and *C. alticolis*), and we did not notice any SNPs for *C. ditrichi*, *C. suis,* and *Cryptosporidium* vole genotype II.

## 4. Discussion

To date, there have only been a limited number of molecular reports regarding the occurrence of *Cryptosporidium* spp. in raccoons from North America [8] and introduced to Poland [10], Japan [12], Germany [13], and Iran [15]. To date, the literature data concerning this issue are mainly based on the reports from the area of North America, where the raccoon is indigenous to the local fauna. *Cryptosporidium* infection has been reported in U.S. raccoons, with the prevalence ranging from 4.0% to 20.5% [7,8,29], of which the genotypes W13 (skunk genotype) and W4 were the characterized genotypes [8,30]. In the present study, the overall prevalence of *Cryptosporidium* spp. was estimated at 24.6%. Molecular analysis revealed the existence of the *Cryptosporidium* spp. skunk genotype. This genotype appears to be the major genotype reported in raccoons from non-native areas [13,14,15]. The majority of the skunk genotype was also reported in Japan [12]. This genotype was reported in 3/5 of the *Cryptosporidium*-positive isolates from these hosts. On the other hand, the study mentioned above resulted in the first report of *C. parvum* in raccoons [12]. It is worth noting that the *Cryptosporidium* skunk genotype has also been reported in a small number of human cases from the U.K. and U.S.A. [30], indicating a zoonotic concern for this genotype [31,32]. The *Cryptosporidium* skunk genotype, as the name suggests, was originally isolated from a striped skunk [6,33]. Prediger et al. (2017) [33] propose that the *Cryptosporidium* skunk genotype may have been introduced to Europe with hosts that are native to North America. The research conducted on *Cryptosporidium* spp. by the authors mentioned above in tree squirrels from Italy has shown that native red squirrels and introduced grey and Pallas’s squirrels host different *Cryptosporidium* spp., with no evidence of transmission between introduced and native hosts [33]. On the other hand, we have detected the *Cryptosporidium* skunk genotype in both non-native to Europe (raccoon) and native (badger) hosts. To the best of our knowledge, this is the first report concerning the *Cryptosporidium* skunk genotype in badgers, whereas the study by Mateo et al. (2017) [11] demonstrated the presence of *C. hominis* in this carnivore host. We consider that the occurrence of the *Cryptosporidium* skunk genotype in badgers studied might be evidence of transmission between introduced and native host species.

Molecular epidemiological data demonstrating the occurrence of *Cryptosporidium* spp. in Canidae, specifically red fox and raccoon dog, derive mostly from research carried out on animals farmed for their fur. There have been relatively few studies that examined the prevalence of these parasites in wild canines [11,14,34]. The raccoon dog, regarded as one of the most successful invasive alien carnivores, has established flourishing, self-sustaining populations in Eastern, Central, and parts of Northern Europe. Its further expansion to the west and south is still in progress [22]. The red fox is the most common wild Canidae species in Europe [35]. These two native and invasive Canidae species contaminate their environment with their pathogens/parasites [36]. Since their home ranges overlap, there are opportunities for their respective infectious agents to switch and adapt to the new host species. Thus, possible bidirectional transmission of microparasites occurring in areas cohabited by these predators, such as the red fox and raccoon dog, may be observed.

Relatively little is known about the distribution of zoonotic and non-zoonotic *Cryptosporidium* species/genotypes in European wild canine populations. For the first time, we examined the occurrence of *Cryptosporidium* spp. in wild populations of the invasive raccoon dog in Europe and added to the data by considering this protozoan parasite in wild foxes. The surveys carried out on foxes in Ireland, Spain, and Eastern European countries, such as the Czech Republic, Slovakia, and Poland, reported the prevalence of *Cryptosporidium* at the level from 2.6% to 8.0% and demonstrated that wild red foxes are suitable hosts for a wide variety of *Cryptosporidium* species, including *C. canis*, *C. felis*, *C. parvum*, *C. ubiquitum, C. hominis,* and *C. suis* [9,11,34], as well as *C. tryzzeri, C. andersoni,* and *C. galii* [14]. *Cryptosporidium canis* is commonly identified in domestic and wild canids worldwide. Cases of human cryptosporidiosis have been recorded in both immunocompetent and immunocompromised persons [1,32,37,38], demonstrating the zoonotic potential of this species. Phylogenetic analysis distinguishes three different genotypes of *C. canis*: dog, fox, and coyote [6]. However, only the *C. canis* dog genotype has been reported as pathogenic in humans [7]. During this study, we found the prevalence of *Cryptosporidium* spp. in foxes to be 12%, consistent with previous reports. We identified three *Cryptosporidium* species/genotypes in our study: *C. canis* fox specific genotype, *Cryptosporidium* vole genotype II, and *Cryptosporidium alticolis* related to those found in common voles [39]. Similarly, in raccoon dogs, apart from the *C. canis* dog-specific genotype, we also observed the presence of *C. suis* and *C. erinacei*, non-specific for this canid and usually isolated from pigs and wild boars, or hedgehogs, respectively. It is interesting that this research has shown wild-living introduced (the raccoon dog) and native (the red fox) canine species are infected with different *Cryptosporidium* genotypes, with no evidence of transmission between introduced and native Canidae. During our survey, we observed the presence of *C. erinacei* DNA in the samples of badgers and *C. ditrichi* in pine and beech martens. This is the first time that these parasite species have been observed in Mustelidae. *Cryptosporidium ditrichi* is characteristic for rodents of the genus *Apodemus* spp., as described in studies conducted on rodent populations from Central European countries [40,41].

The diet of these wild carnivores may explain the anomalous presence of these *Cryptosporidium* spp. Red foxes act as natural scavengers in their habitats and could harbor non-specific cervid cryptosporidial oocysts, providing a further route for their dissemination [42,43]. The ordinary diet of red foxes includes insects, birds, and small mammals; since *Cryptosporidium* spp. oocysts are routinely found in small rodents [39,40,44], the presence of rodent-specific cryptosporidia in foxes is not surprising. Similar results were reported by Kváč et al. (2021) [14], demonstrating the presence of non-specific *C. tryzzeri, C. andersoni,* and *C. galii* in foxes. Raccoon dogs consume a broadly omnivorous diet, consisting mainly of small rodents, birds, amphibians, and occasionally reptiles, invertebrates, fruits, and cereals [22,45,46]. *Cryptosporidium suis* has been isolated from Eurasian wild boars but also from rodents [47,48]. Castro-Hermida et al. (2011) [49] have suggested that wild boars contaminate surface water with *Cryptosporidium* oocysts because their activities close to streams and marshy areas facilitate the circulation of this enteropathogen. Thus, the observation of *C. suis* in raccoon dogs can be explained by the presence of swine cryptosporidia in source water [50,51]. Little is known about the epidemiology or pathogenicity of the zoonotic *C. erinacei* in wildlife. The finding that at least 30% of European hedgehogs shed oocysts of *C. parvum* or *C. erinacei* contributes to environmental contamination with these protozoan parasites [52]. Past studies of wild raccoon dogs found them to harbor only *C. canis*; our study provides the first record of raccoon dogs as reservoir hosts of *C. erinacei* and *C. suis.* This is especially important since *C. suis* has been reported to be pathogenic in humans [53].

The interesting question emerging from our finding of non-specific *Cryptosporidium* spp. in examined canids and mustelids is whether the respective oocysts merely pass through the digestive tract of the animals or whether these carnivores are infected with these non-specific *Cryptosporidium* spp. and subsequently excrete their oocysts.

In our survey, we used molecular methods to identify and genotype *Cryptosporidium* spp. in wild-living carnivore species co-occurring in the same area (native and introduced species) and assessed these mesocarnivores as a significant reservoir of *Cryptosporidium* species, including those with zoonotic potential. We understand this study might have some limitations regarding the samples’ geographical origin and applied tools. Because of the fact that the sequencing was performed for all actin amplicons and some of the 18S rRNA gene, there is the possibility of non-specific amplifications, which might be expected because the 18S rRNA gene is conserved among different species. We performed the nested PCRs targeting the 18S rRNA gene as screening and detection method. Then, we sequenced selected 18S rRNA products to confirm the specific amplification of *Cryptosporidium*. As shown by Sulaiman et al. (2002) [27], the actin gene, due to its genetic variability, is a good phylogenetic marker for analysis of taxonomic relationships of *Cryptosporidium* parasites. Importantly, the results from the actin gene validated observations on the multispecies nature of *Cryptosporidium* based on sequence analysis of the 18S rRNA gene [27]. As shown by results obtained by other authors conducting research on fecal samples from wild-living hosts [14,54,55], the genetic relationship among various *Cryptosporidium* parasites revealed by phylogenetic analysis of the actin gene is largely in agreement with conclusions based on the results from 18S rRNA.

Further studies are needed to acquire more sequence data from *Cryptosporidium* isolates from diverse native and non-native hosts and different geographical areas. This can lead to a comprehensive understanding of the genetic diversity, host specificity, and transmission dynamics of *Cryptosporidium* in order to better understand the likelihood of wild carnivores representing a public health risk for transmission of *Cryptosporidium*. The use of next generation MLST tools will further improve our understanding of the epidemiology of cryptosporidiosis.

## 5. Conclusions

This study is one of the few to assess the significance of wild-living carnivores (native and introduced) in the epidemiology of *Cryptosporidium* spp. Molecular data presented here indicate that mesocarnivores belonging to Procyonidae, Canidae, and Mustelidae living in the wild in Poland may be a significant reservoir of specific and non-specific *Cryptosporidium* species and genotypes, including those with zoonotic potential. Most studies on carnivores have described the presence of non-specific *Cryptosporidium* spp. in these hosts and is most likely the result of the transfer of these parasites from prey species through the digestive tract or transfer of the parasite from a contaminated environment.

## Figures and Tables

**Table 1:** Prevalence and genotyping of *Cryptosporidium* spp. in raccoons, raccoon dogs, red foxes, badgers, and martens obtained in this study.

Carnivores/Host Species (Common Name)		No. Examined/No. Positive Samples (%; 95% Confidence Interval)	Genotyping *Cryptosporidium* spp. Actin Locus (No. of Sequences Samples)
Procyonidae	*Procyon lotor* (raccoon)	65/16 (24.6; 16.2–35.4)	*Cryptosporidium* skunk genotype (16)
Canidae	*Nyctereutes procyonoides* (raccoon dog)	87/21 (24.1; 14.5–36.9)	*Cryptosporidium canis* (dog genotype) (16) *Cryptosporidium erinacei* (3) *Cryptosporidium suis* (2)
	*Vulpes vulpes* (red fox)	50/6 (12.0; 4.7–22.8)	*Cryptosporidium canis* (fox genotype) (3) *Cryptosporidium alticolis* (2) *Cryptosporidium* vole genotype II (1)
Mustelidae	*Meles meles* (badger)	45/9 (20.0; 9.5–37.1)	*Cryptosporidium* skunk genotype (5) *Cryptosporidium erinacei* (4)
	*Martes martes* (pine marten)	24/7 (29.2; 13.9–50.0)	*Cryptosporidium ditrichi* (7)
	*Martes foina* (beech marten)	51/15 (29.4; 20.8–39.5)	*Cryptosporidium ditrichi* (15)
	Total	322/74 (23.0; 18.7–27.9)	

## Data Availability

The data presented in this study are available on request from the corresponding author.

## Supplementary Materials

The following supporting information can be downloaded at: https://www.mdpi.com/article/10.3390/pathogens12020198/s1, Figure S1: M-DNA mass marker (DNA Ladder, EURx), 235–258 are tested samples, K^−^—negative control (with distilled water), K^+^—positive control.; Figure S2: M-DNA mass marker (DNA Ladder, EURx), PL80, PL81, PL85, PL92, PL94, PL95, PL69, 36 are tested samples, K^−^—negative control (with distilled water), K^+^—positive control.
