# Supplementary material for: The Occurrence of Cryptosporidium spp. in Wild-Living Carnivores in Poland—A Question Concerning Its Host Specificity
Source: Pathogens. 2023 Jan 28;12(2):198. doi: 10.3390/pathogens12020198 (PMC9968153; doi:10.3390/pathogens12020198)
Supplement: Supplementary file 1 [file pathogens-12-00198-s001.zip › pathogens-2054003-supplementary.pdf]

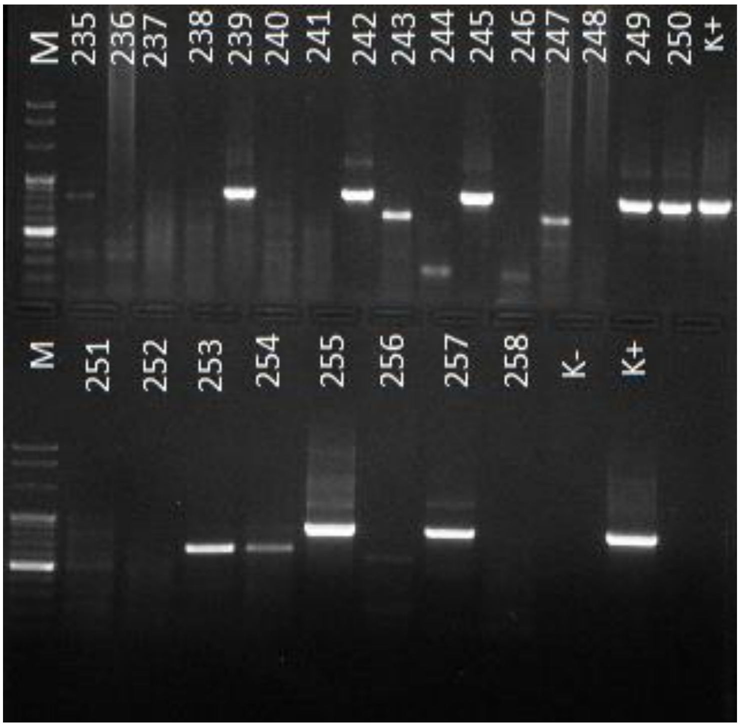

Figure S1. M-DNA mass marker (DNA Ladder, EURx), 235-258 are tested samples, K- - negative control (with distilled water), K+ - positive control. Electrophoregram presenting study results – *Cryptosporidium* spp. (18S rRNA as target; 819 to 825 bp).

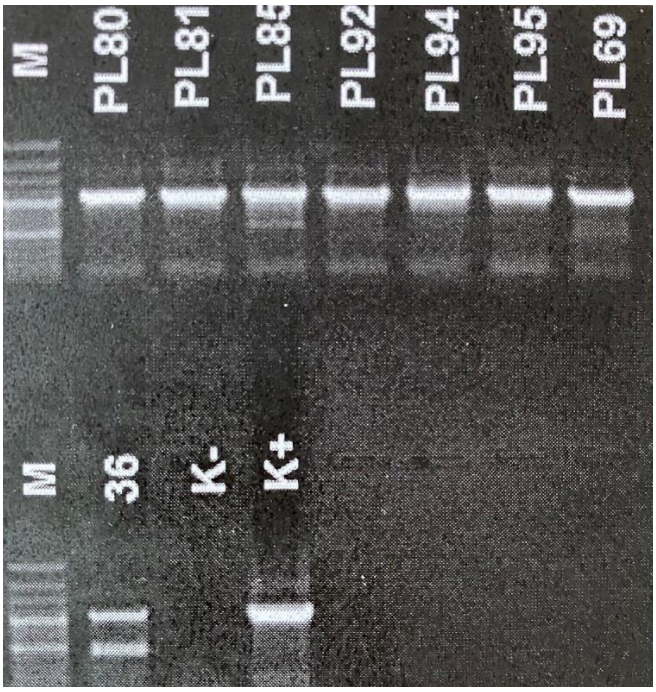

Figure S2. M-DNA mass marker (DNA Ladder, EURx), PL80, PL81, PL85, PL92, PL94, PL95, PL69, 36 are tested samples, K- -negative control (with distilled water), K+ - positive control. Electrophoregram presenting study results – *Cryptosporidium* spp. (actin gene as target; ~1,066 bp).
